# Selective Detection of Carbon Monoxide on P-Block Doped Monolayers of MoTe_2_

**DOI:** 10.1021/acssensors.1c02246

**Published:** 2022-01-19

**Authors:** Maciej J. Szary, Dominik M. Florjan, Jakub A. Bąbelek

**Affiliations:** Institute of Physics, Poznan University of Technology, ul. Piotrowo 3, 61-138 Poznan, Poland

**Keywords:** CO, CO_2_, MoTe_2_, transition metal dichalcogenide, gas sensing, density functional theory

## Abstract

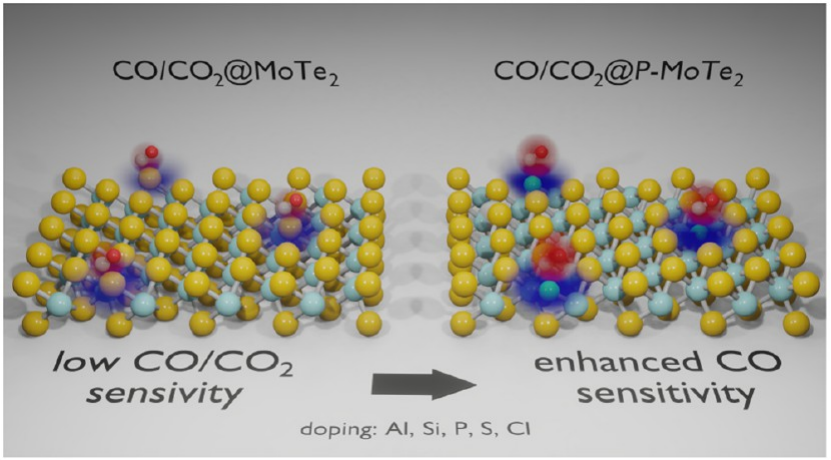

CO and CO_2_ are among the most commonly monitored gases. However, the currently available semiconductor sensors require heating to ∼400 °C in order to operate effectively. This increases the power demand and shortens their lifespan. Consequently, new material prospects are being investigated. The adoption of novel two-dimensional layered materials is one of the pursued solutions. MoS_2_ and MoTe_2_ sheets have already been shown sensitive to NO_2_ and NH_3_ even at room temperature. However, their response to other compounds is limited. Hence, this work investigates, by employing density functional theory (DFT) calculations, the doping of Al, Si, P, S, and Cl atoms into the Te vacancy of MoTe_2_, and its impact on the sensing characteristics for CO and CO_2_. The computations predict that P doping significantly enhances the molecule-sheet charge transfer (up to +436%) while having only a little effect on the adsorption energy (molecular dynamics show that the molecule can effectively diffuse at 300 K). On the other hand, the doping has a limited impact on the adsorption of CO_2_. The relative (CO/CO_2_) response of P-doped MoTe_2_ is 5.6 compared to the 1.5 predicted for the pristine sheet. Thus, the doping should allow for more selective detection of CO in CO/CO_2_ mixtures.

Carbon dioxide (CO_2_) and carbon monoxide (CO) are the principal products of combustion of fossil fuels. Hence, their emissions are extremely prevalent in transportation, electricity production, industrial processes, as well as commercial and residential applications.^1−4^ Despite that, both gases can be harmful to human health, with CO being significantly more dangerous than CO_2_.^5,6^ CO is formed when carbon in fuel is not burned completely, which makes the optimal supply of oxygen (O_2_) doubly important. The health effects of CO depend on its concentration and the length of exposure. However, early symptoms of prolonged exposure can occur at concentrations as low as 35 ppm, while 3200 ppm can cause death within 30 min.^7,8^ Consequently, CO sensors are required in various situations, and thus the detection methods are constantly perfected.^9−14^ At the same time, new prospects are investigated,^15−19^ among which novel nanomaterials have gained noticeable interest.^20−25^

Transition-metal-dichalcogenide (TMD) sheets are a class of two-dimensional (2D) layered materials. Their chemical formula is MY_2_, and they consist of one transition-metal layer (M) sandwiched between two chalcogen layers (Y). The elements within these three layers bond covalently, while individual three-layer sheets interact with each other via weak van der Waals (vdW) forces, which allows for their effective exfoliation.^26,27^ TMDs have gained a lot of interest in recent years due to their unique properties like tunable band gap,^28−30^ high carrier mobility,^31,32^ and strong spin–orbit coupling,^33^ to name a few. These properties make them compelling platforms for novel applications in fields such as electronics,^34−36^ energy storage,^37−39^ and medicine.^40,41^ Particularly engrossing is the use of the sheets in gas sensing. A small amount of sensing material and the excellent surface-to-volume ratio combined with the sizable band gap of some TMDs (e.g., MoY_2_ and WY_2_) could make them into a high-sensitivity low-cost alternative to metal-oxide-semiconductor (MOS) devices. Especially considering the promising reports on the effective detection at room temperature achieved by TMD-based sensors,^42−45^ which is known to be problematic for MOS-type devices.^46−49^

TMDs such as MoTe_2_ and MoS_2_ have been found to be sensitive toward molecules of nitrogen dioxide (NO_2_)^50−53^ and ammonia (NH_3_).^53−56^ However, the response of their sheets toward other compounds is relatively low due to the weak interactions on their surface. This limits the number of analytes compatible with pristine TMDs. Hence, a significant effort has been put into the modification of TMDs to enhance their sensitivity toward selected molecules.^57−59^ In the most common approaches, the sheets have been decorated with nanoparticles^60−62^ or single atoms,^63^ or they have been doped with single-atom impurities.^39,64−68^ Doping has been shown especially effective in enhancing the values of adsorption energy and charge transfer for nitrogen^69−72^ and sulfur-containing gases,^72−74^ as well as other compounds including formaldehyde (CH_2_O),^66,68^ ethylene oxide (C_2_H_4_O),^75^ and histamine (C_5_H_9_N_3_).^76^ However, the reported values often indicated that the doping facilitated strong chemisorption. Such interactions could impede the recovery rate of the adsorption site and thus hinder the response to fast changes in concentration.^51^ Hence, an optimal doping strategy for gas detection would (i) increase the values of the charge transfer in the vicinity of the doping site, such that (ii) the effect would enhance the intrinsic transfer of the sheet without (iii) a significant increase in the adsorption energy. Consequently, this work investigates employing density functional theory (DFT) calculations, the impact of Al, Si, P, S, and Cl doping of MoTe_2_ on binding, and charge transfer for CO and CO_2_ adsorption, and the extent to which it may enhance their detection.

## Computational Details

The computational results included in this report were obtained using the Quantum ESPRESSO package.^77−79^ The visualizations of atomic structures were created using the XCrySDen program.^80^ Rappe–Rabe–Kaxiras–Joannopoulos ultrasoft-type pseudopotentials^81^ were used. Generalized gradient approximation (GGA) with Perder-Burke-Ernzerhof (PBE) parametrization^82,83^ was adopted to treat the electron exchange and correlation. VdW contributions to the total energy were treated using Grimme’s DFT-D3 method.^84^ Energy cutoffs were set to 50 and 500 Ry for the wave function and density, respectively. The smearing parameter was set to 0.01 Ry. The *k*-points were generated from 12 × 12 × 1 mesh using the Monkhorst–Pack method.^85^ Ab initio molecular dynamics (AIMD) calculations were based on Born–Oppenheimer theory. Verlet approximation was used for the integration of equations of motion. All parameters were kept except for Monkhorst–Pack grid, which was reduced to 4 × 4 × 1. The time step was set at 20 au (0.9676 fs) with the target temperature set at 300 K. The charge transfers were calculated as differences in charge obtained from Löwdin population analysis for pseudoelectron density, i.e., the valence electron density. Consequently, the core electrons were not included, and only the net changes were investigated.

MoTe_2_ monolayer was modeled by a 2D periodic slab. 3 × 3, 4 × 4, and 5 × 5 cells were tested for doping and molecule adsorption. The computations have shown that the structure optimizations done in 4 × 4 and 5 × 5 cells have resulted in nearly identical doping and adsorption energies, while the values for 3 × 3 were noticeably different. Thus, the 4 × 4 unit cell was employed in the following computations. Cell height of 20 Å was adopted in order to minimize artificial interactions between the neighboring systems. Atomic positions of all atoms in the system were relaxed in the total energy optimization with the adopted convergence threshold on forces of <10^–8^ Ry/au.

## Results and Discussion

### Doping of MoTe_2_

**Figure 1:**
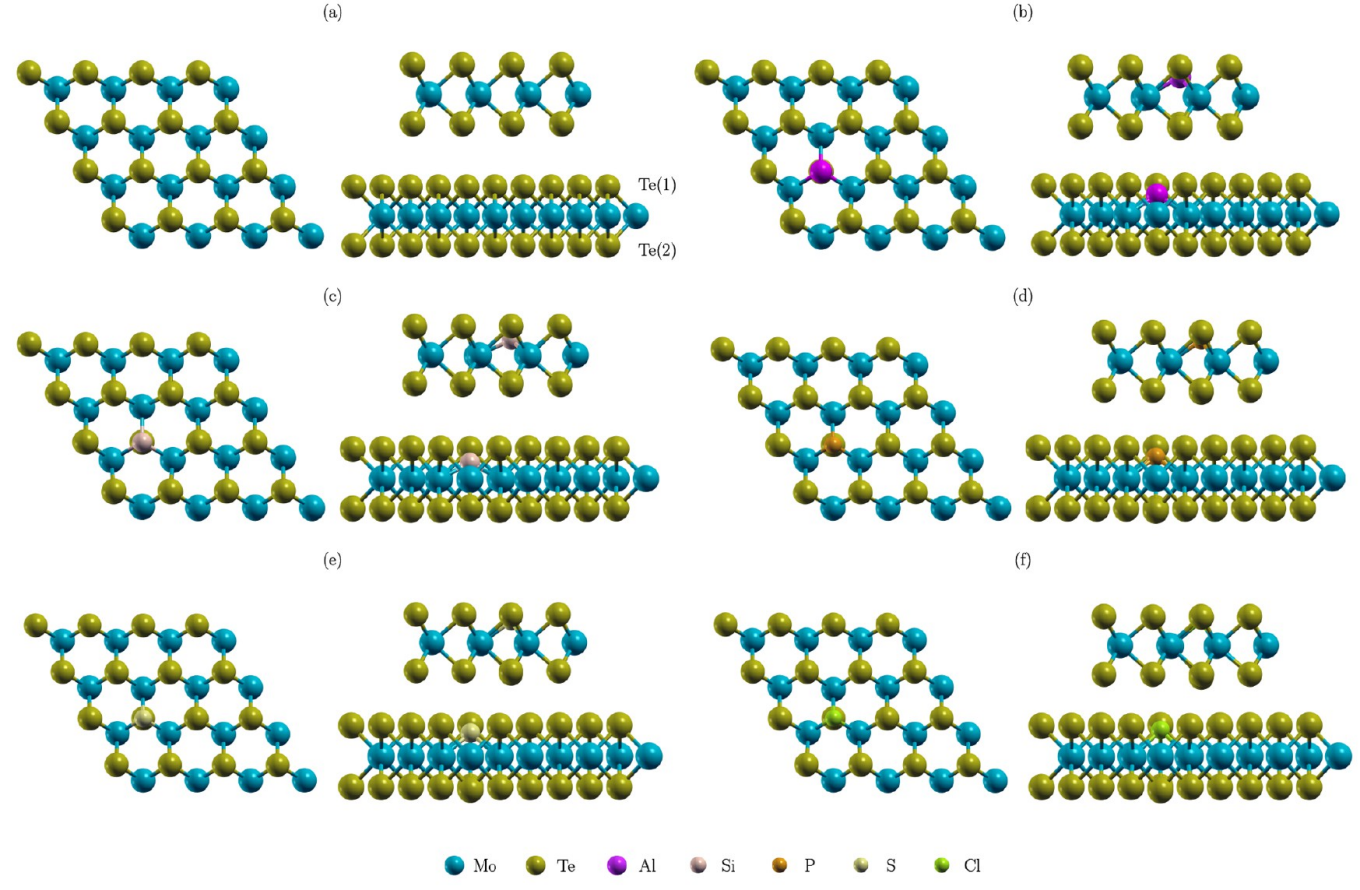
Schematics of atomic structures of MoTe_2_ (a), Al-MoTe_2_ (b), Si-MoTe_2_ (c), P-MoTe_2_ (d), S-MoTe_2_ (e), and Cl-MoTe_2_ (f).

This work investigates, with the use of computational methods, doping strategies for the enhanced sensitivity of MoTe_2_ toward combustion products CO and CO_2_. The employed doping model was based on the experimental findings of Yang et al.,^86^ where the authors reported on large-sized single-crystal sheets of MoTe_2_ grown using the CVD method with up to 10% of Te atoms substituted by S. The sheets were modeled with 2DPS employing a 4 × 4 unit cell of MoTe_2_, comprising 48 atoms. Other cell sizes have also been tested (see the description given in Computational Details). Doping was done by removing one tellurium atom per supercell from the upper layer of MoTe_2_ [Te(1)] and replacing it with a dopant (X); see Figure 1. This results in ∼2.1% doping concentration, which should make such structures feasible to fabricate employing the same CVD growth methods as previously reported. However, to further increase the viability of the modeled structures, this investigation limits its analyses to the effects introduced by doping with elements of the same period and block as sulfur, namely, Al, Si, P, S, and Cl. This approach should maximize the feasibility of fabrication, while also providing a number of secondary benefits. First, p-block doping typically results in lower adsorption energy of small molecules compared to the effects facilitated by d-block elements.^65,66,75^ Second, the selected elements have their covalent radius smaller than that of Te, which should promote the dopant relaxation in the Te vacancy of MoTe_2_ below the layer of Te(1). This may favor horizontal adsorption near the doping site, which in turn could promote a more selective nature of the facilitated effects due to the different lengths of the molecules.

The optimized structures of pristine and doped MoTe_2_ are illustrated in Figure 1. In all cases, the dopants relax centered between neighboring Mo with relatively short X–Mo distances (2.388–2.552 Å) and a limited impact on the structure of MoTe_2_. The dopants favor positions below the layer of Te(1). However, the shift depends on the element. In order to quantify the effect, we define *Δh*_Te-X_ as the relative difference in height between atoms of Te(1) and X i.e., *Δh*_Te-X_ = *h*_Te_ – *h*_X_. Hence, *Δh*_Te-X_ is positive when X relaxes below Te(1). The values of *Δh*_Te-X_ are summarized in Table 1. The results show a partial correlation of *Δh*_Te-X_ with the number of valence electrons of X. Al and Si (groups 13 and 14) promote high values of *Δh*_Te-X_. Thus, the dopants favor interaction with the neighboring Mo of a strong in-plane character. On the other hand, P, S, and Cl (groups 15–17) facilitate lower values of *Δh*_Te-X_, which indicates that those elements favor the X–Mo bonding with a stronger out-of-plane component. We quantify the strength of X–Mo bonding interaction with the binding energy defined as follows:
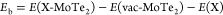
1where *E*(X-MoTe_2_), *E*(vac-MoTe_2_), and *E*(X) are the total energies of the X-doped MoTe_2_, Te-vacancy MoTe_2_, and a free atom of X, respectively. The corresponding values are given in Table 1. The strongest binding is predicted for S with *E*_b_ = −7.338 eV, followed by P and Si. The remaining dopants, Al and Cl, give rise to noticeably weaker binding of ∼ −4 eV. Still, regardless of the element, the values predicted are large indicating the formation of strong chemical bonds upon adsorption of X into the Te vacancy of MoTe_2_.

**Table 1:** Parameters of Optimized Doped Monolayers

	*d*_X-Mo_^a^	*Δh*_X-Te_^b^	–*E*_b_^c^	*ΔQ*_X_^d^	*δ Q*_X-s_^e^	*δ Q*_X-p_^e^	*δ Q*_X-p_*z*__^e^	*δ Q*_X-p_*x*__^e^	*δ Q*_X-p_*y*__^e^
Al-MoTe_2_	2.552	0.721	4.077	–0.131	0.699	–0.83	–0.03	–0.4	–0.4
Si-MoTe_2_	2.43	0.94	6.368	–0.068	0.514	–0.582	0.094	–0.338	–0.338
P-MoTe_2_	2.388	0.397	7.18	–0.164	0.315	–0.479	–0.152	–0.164	–0.164
S-MoTe_2_	2.41	0.275	7.338	–0.201	0.201	–0.401	–0.244	–0.079	–0.079
Cl-MoTe_2_	2.478	0.066	3.91	–0.077	0.121	–0.199	–0.107	–0.046	–0.046

a *d*_X-Mo_ is the distance between dopant and the neighboring Mo atoms (given in Å).

b *Δh*_X-Te_ is the shift in position of X upon substitution of Te (given in Å).

c *E*_b_ is the binding energy (eq 1, given in eV).

d *ΔQ*_X_ is the charge accumulated in X after doping.

e *δQ* represents change in population of molecular orbitals described in subscript (given in elemental charge *e*, i.e., the electric charge carried by a single proton).

**Figure 2:**
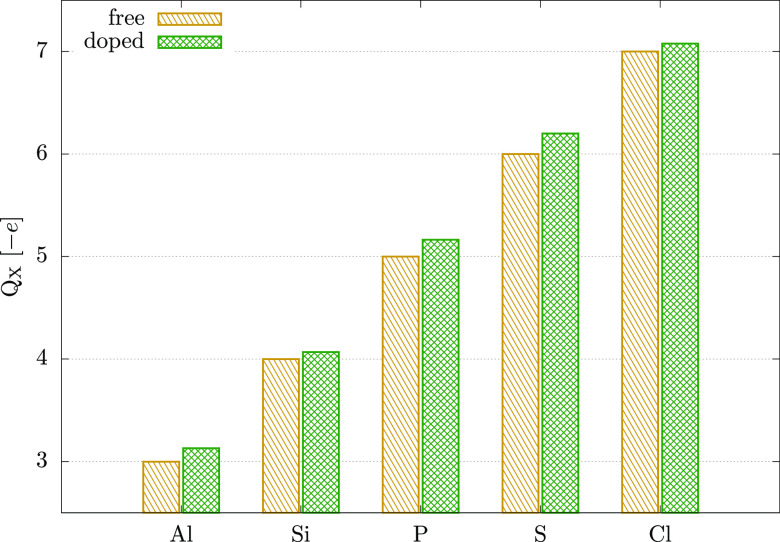
Löwdin orbital population of a free atom and after doping. Only valence electrons are taken into account.

The X–Mo bond formation gives rise to new hybrid states comprising the atomic orbitals of both X and Mo. This in turn can facilitate an effective electron transfer to or from the dopant. Figure 2 shows the total electronic pseudocharge (valence electrons only) of free and adsorbed X. It is predicted that in all investigated cases the dopant gains partial electronic charge upon doping. The values of the accumulated charge *ΔQ*_X_ = *Q*_X_(doped) – *Q*_X_(free) are given in Table 1. S and P gain the most electronic pseudocharge, which coincides with their strong binding. However, Al also facilitates a relatively high *ΔQ*_X_ despite a significantly weaker interaction. On the other hand, it is evident that the predicted values are a product of s orbital depopulation (*δQ*_X-s_) and an electron gain in the p subshell *δQ*_X-p_ (see Table 1), and also that the differences become smaller for elements with more electrons.

In order to accommodate the structurally imposed geometry of the X–Mo interaction, the doped atoms have to adopt hybrids suitable for a trigonal-pyramidal bonding, i.e., sp^λ^ (2 < λ ≤ 3). Hence, some of the differences shown between the dopants may be a product of hybridization. The depopulation of s orbitals is in line with sp-type hybrids, so the main difference should arise in the p subshell. In the case of Al and Si, p_*x*_ and p_*y*_ orbitals gain most of the accumulated charge, while the s orbital gains little (Al) or even loses electrons (Si). This indicates that the atoms favor interaction with a strong in-plane character, which coincides with the high values of *Δh*_Te-X_. Consequently, hybrids with λ < 3 are expected. However, λ values should still be larger than 2, as the bonding geometry is trigonal-pyramidal rather than planar. This in turn makes Si (group 13 element) in need of electrons to fill the hybrid states and thus promotes an enhanced electron accumulation. In contrast, P, S, and Cl gain less charge in their p_*x*_ and p_*y*_ orbitals, while accumulating more charge in p_*z*_. This suggests an X–Mo interaction with a stronger out-of-plane component, hence a hybridization with a higher value of λ compared to Al and Si.

**Figure 3:**
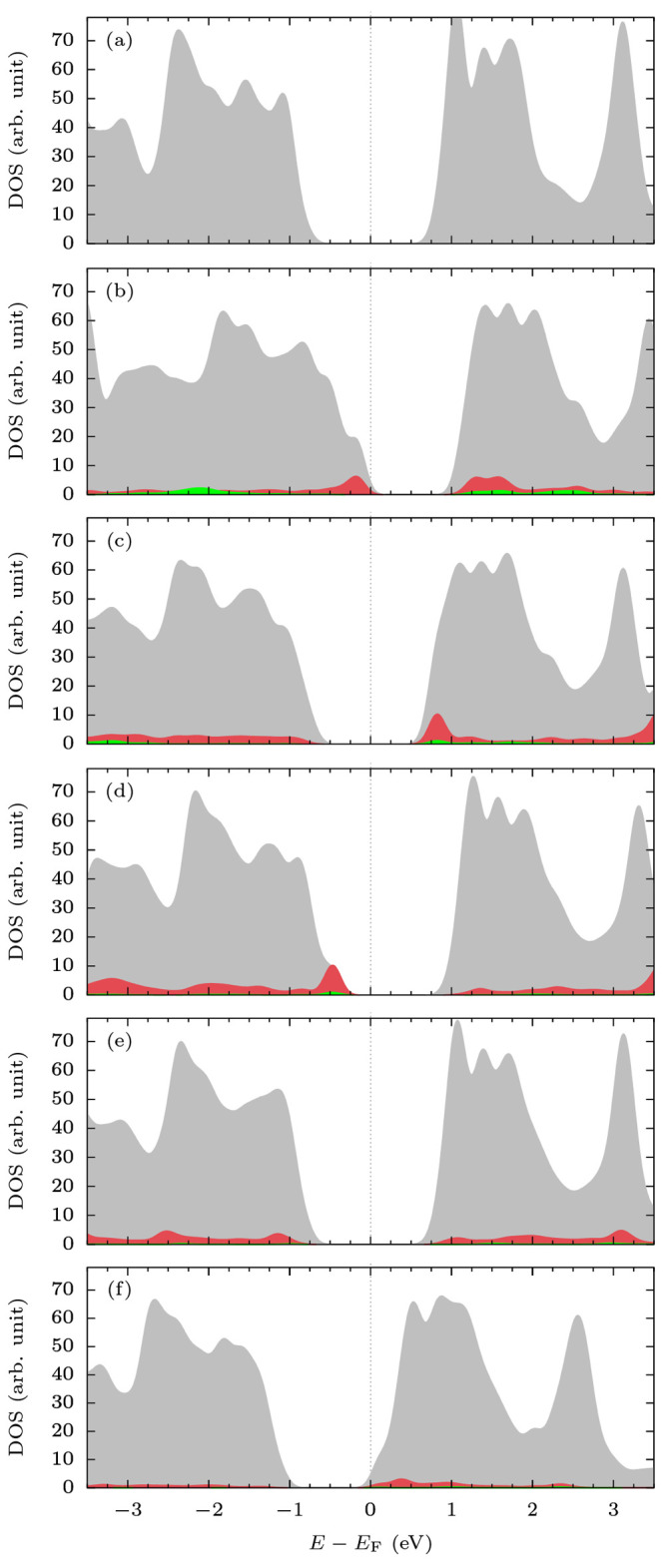
DOS contours of (a) MoTe_2_, (b) Al-MoTe_2_, (c) Si-MoTe_2_, (d) P-MoTe_2_, (e) S-MoTe_2_, and (f) Cl-MoTe_2_. Gray contours represent the total DOS, while red and green the partial DOS of p and s orbitals of X. The values of partial DOS have been multiplied by a factor of 5 to highlight the distribution of impurity states.

Covalent functionalization can have a significant impact on the band structure of TMDs, which in turn determines the gas-sensing mechanism of a sensor.^70,87,88^ Hence, it is crucial to ascertain the changes in the electronic properties of MoTe_2_ after doping. Figure 3a shows the total density of states (DOS) of MoTe_2_, while Figure 3b–f illustrates the total DOS of the doped sheets (gray) and the partial DOS of p (red) and s (green) orbitals of X. Furthermore, Table 2 summarizes the values of the work function (*W*) and the electronic band gap (*E*_g_) of the pristine and doped layers.

**Table 2:** Work Functions and Band Gaps of Pristine and Doped

	work function^a^ (eV)	band gaps (eV)
MoTe_2_	4.571	1.06
Al-MoTe_2_	5.042	metallic
Si-MoTe_2_	4.613	0.85
P-MoTe_2_	4.833	0.83
S-MoTe_2_	4.669	0.97
Cl-MoTe_2_	4.219	metallic

a Work function is given by *W* = *V*(vac) – *E*_F_, where *V*(vac) and *E*_F_ are electrostatic potential in a vacuum region far from the sheet and the Fermi energy of the system, respectively.^88^

Monolayer MoTe_2_ is predicted to be a semiconductor with a band gap of 1.05 eV and a work function of 4.571 eV. Doping with Al (group 13) moves down the Fermi level (*E*_F_) due to its lower number of valence electrons compared to Te (group 16). Consequently, the work function increases to 5.042 eV, and the sheet transitions from semiconducting to a metallic system after a complete band gap reduction (see Figure 3b). The gap closing coincides with the metallic bands comprising the p orbitals of Al. In the case of Si-MoTe_2_, more valence electrons in Si make the impact of doping less pronounced (see Figure 3c). The sheet remains a semiconductor with a reduced band gap of 0.85 eV. The doping facilitates only a small change in the Fermi energy. Hence, it has no significant effect on the work function and the dominant character of majority charge carriers (n-type or p-type).

Interestingly, P doping has a similar impact on the electronic properties of the sheet as Al (see Figure 3b and d), despite having more valence electrons. We ascribe this to the higher-λ hybrids predicted for P, S, and Cl dopants. Doping with P decreases the Fermi energy of the system, which increases its work function to 4.833 eV. However, unlike Al-MoTe_2_, the P-doped sheet remains semiconducting with a band gap of 0.85 eV and a p-type conduction. The reduction of *E*_g_ is a result of new electronic states, which occupy the top of the valence band and comprise the orbitals of P. In the case of S-MoTe_2_ (Figure 3e), the dopant has the same valence configuration as Te. Hence, the doping is predicted to have a limited effect on the electrical properties of the sheet. The system remains semiconducting with a band gap of 0.97 eV. Furthermore, its Fermi energy is reduced by ∼0.1 eV, which results in a small increase in its work function. Finally, the DOS of Cl-MoTe_2_ is shown in Figure 3f. Cl is the only dopant with more valence electrons than Te. Hence, it is the only element that shifts Fermi energy higher than the pristine MoTe_2_, i.e., electron doping the sheet. This in turn results in a complete band gap reduction.

### Adsorption of CO on Pristine and Doped MoTe_2_

The interactions between molecules and TMD monolayers are typically limited to vdW forces,^27,28,89^ coming from the low chemical activity of the sheet. Because of that, the adsorption lacks one well-defined site as the molecules can relax into a number of different orientations.^51,74,90^ The configurations often share relatively similar adsorption parameters due to the dispersive nature of the dominant interaction mechanism. However, they are separated by low activation barriers, and thus transitions between semistable states are common even at relatively low temperatures. Hence, in order to provide a more detailed depiction of the interaction between CO and MoTe_2_/X-MoTe_2_, this study employs four different initial adsorption geometries: (i) CO placed vertically with O atom above Te/X (vert-O, Figure S1a), (ii) vertically with C atom above Te/X (vert-C, Figure S1b, see Supporting Information), (iii) horizontally with O atom above Te/X (horiz-O, Figure S1c), and (iv) horizontally with C atom above Te/X (horiz-C, Figure S1d).

In order to improve the detection characteristics of a TMD, the doping has to increase the value of the charge transfer between the analyte and the monolayer in the vicinity of the doping site such that, the effect would enhance the intrinsic transfer of the sheet without a significant increase in the adsorption energy. Hence, to quantify the effects, we introduce the adsorption energy *E*_ads_, the corresponding CO–substrate charge transfer *ΔQ*_CO_, and the difference in charge accumulation in the dopant *ΔQ*_X_^ads^. The adsorption energy is defined as
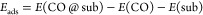
2where *E*(CO@sub) is the total energy of the adsorbate–substrate system (MoTe_2_ or X-MoTe_2_), *E*(CO) is the energy of free molecule, and *E*(sub) is the monolayer. Consequently, *E*_ads_ is positive for endothermic and negative for exothermic processes. The CO-substrate charge transfer follows the formula:
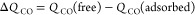
3where *Q*_CO_ is the total pseudocharge (a sum over all orbitals and atoms) of the free and adsorbed molecule. Hence, *ΔQ*_CO_ represents the total depopulation of CO, and thus also the charge transfer into the sheet facilitated upon adsorption. The difference in charge accumulation introduced by the adsorption in defined as
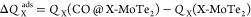
4where *Q*_X_(X-MoTe_2_) and *Q*_X_(CO@*X*-MoTe_2_) are the total pseudocharge of X pre- and post-adsorption, respectively. Its value gives the electronic accumulation in the dopant facilitated by the interaction between CO and X.

**Figure 4:**
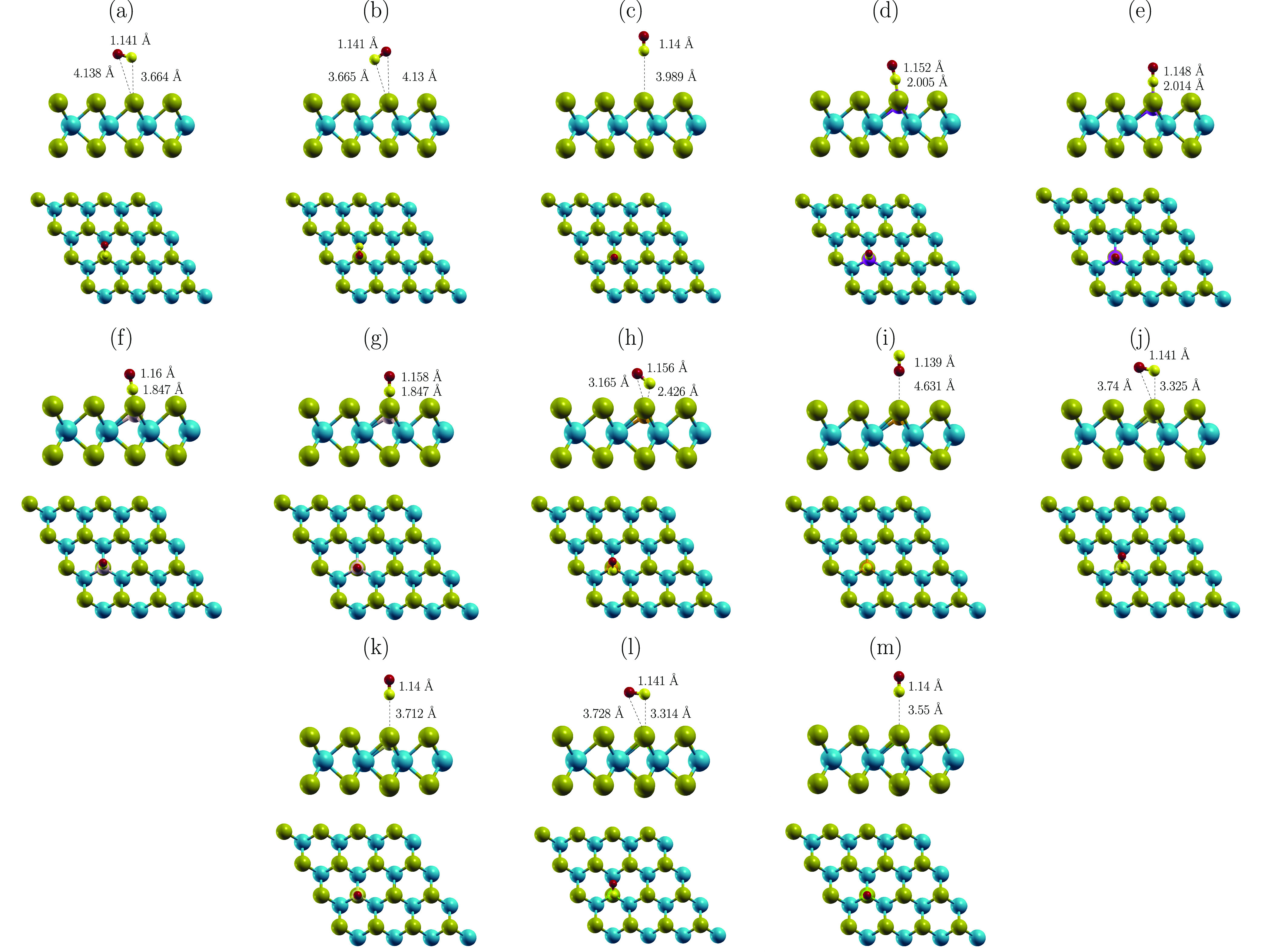
Schematics of optimized structures of CO on (a) MoTe_2_ (horiz-C), (b) MoTe_2_ (horiz-O), (c) MoTe_2_ (vert-C), (d) Al-MoTe_2_ (horiz-C), (e) Al-MoTe_2_ (vert-C), (f) Si-MoTe_2_ (horiz-C), (g) Si-MoTe_2_ (vert-C), (h) P-MoTe_2_ (horiz-C), (i) P-MoTe_2_ (vert-O), (j) S-MoTe_2_ (horiz-C), (k) S-MoTe_2_ (vert-C), (l) Cl-MoTe_2_ (horiz-C), and (m) Cl-MoTe_2_ (vert-C).

The adsorption parameters of every investigated structure are summarized in Table 3, while selected configurations are shown in Figure 4. In the case of pristine MoTe_2_, the molecule of CO relaxes at a relatively large distance from the substrate. After the optimization, the vertical configurations show a noticeable tilt of the molecule relative to the surface with the lower and upper atoms of CO at approximately 3.7 and 4.1 Å distance from the nearest-neighbor Te, respectively (see Figure 4a and b). On the other hand, the vertical configurations promote virtually identical structures (differing only in the C/O orientation), with the molecule-sheet distance of ∼4 Å (configuration vert-C shown in Figures 4c). The adsorption has also almost no impact on the molecule and the substrate. The preadsorption C–O distance was computed to be 1.1398 Å, while its post-adsorption values are 1.140–1.141 Å depending on the configuration. This is also the case for the Te–Mo distance in the vicinity of the adsorption site, where the values change from 2.733 to ∼2.79 Å. The large molecule-sheet distances and the small changes in their atomic structure suggest a physisorption of CO, which is in line with the predicted values of *E*_ads_. The adsorption energy depends on the configuration (see Table 3). However, it is −113 meV or less, where the minus indicates an exothermic process, i.e., a case where it is energetically favorable for the molecule to adsorb rather than both systems remaining isolated. The weak adsorption coincides with the low charge transfer facilitated between CO and MoTe_2_ (see Table 3). The computations predict that no more than 0.024 *e* is transferred from the molecule to the substrate, which makes the sheet relatively insensitive toward CO, when compared to a molecule such as NO_2_ where the maximum transfer is 0.034–0.06 *e*.^90^ However, the computations show that the doping can enhance the adsorption parameters for the molecule.

**Table 3:** Adsorption of CO on MoTe_2_ and X-MoTe_2_

	conf.	Figure	–*E*_ads_^a^	*ΔQ*_CO_^b^	*ΔQ*_X_^ads^^c^
MoTe_2_	horiz-C	4a	103	0.023	-
	horiz-O	4b	113	0.024	-
	vert-C	4c	66	0.003	-
	vert-O		61	0.001	-
Al-MoTe_2_	horiz-C	4d	957	–0.039	–0.066
	horiz-O		187	0.073	–0.018
	vert-C	4e	911	–0.053	–0.076
	vert-O		192	–0.039	–0.013
Si-MoTe_2_	horiz-C	4f	1047	0.023	0.029
	horiz-O		171	0.014	–0.011
	vert-C	4g	1047	0.018	0.026
	vert-O		156	–0.014	0.005
P-MoTe_2_	horiz-C	4h	234	0.128	0.122
	horiz-O		220	0.099	0.074
	vert-C		115	0.001	0.040
	vert-O	4i	95	0	0.020
S-MoTe_2_	horiz-C	4j	151	0.029	0.013
	horiz-O		156	0.034	0.011
	vert-C	4k	94	0.006	0.019
	vert-O		88	0.002	0.011
Cl-MoTe_2_	horiz-C	4l	128	0.019	0.007
	horiz-O		131	0.021	0.003
	vert-C	4m	104	0.007	0.023
	vert-O		92	0.003	0.012

a *E*_ads_ is the adsorption energy (meV)

b *ΔQ*_CO_ is the change in the total charge of the molecule upon its adsorption (given in *e*)

c *ΔQ*_X_^ads^ is the change in the total charge in X upon adsorption of CO (given in *e*).

Al and Si dopants promote the strongest adsorption of CO among the investigated elements. In both cases, the doping results in relatively similar structures and the corresponding adsorption parameters. Al and Si strongly favor configurations with the C atom facing the dopant. In those cases, the molecule relaxes vertically regardless of the initial geometry (see Figure 4d–g). The C–Al and C–Si distances are 2.01 and 1.85 Å, respectively. Both values are significantly shorter than 3.66 Å predicted for the pristine sheet. The adsorption on Al-MoTe_2_ and Si-MoTe_2_ is also shown to have a slightly larger impact on the C–O bond length compared to MoTe_2_. The post-adsorption C–O distance is 1.148–1.16 Å on the doped sheet, while it is ∼1.41 Å on the pristine MoTe_2_. The adsorption results in *E*_ads_ between −0.911 and −1.047 eV. In contrast, the configurations with O facing the dopant are similar to the adsorption on the pristine MoTe_2_. The O–X distances are 2.831–3.317 Å, while the energy of the interaction is less than 200 meV. The charge transfer computed for Al-MoTe_2_ shows notable effects introduced by doping. On average, the absolute values are larger than those for the pristine sheet. Furthermore, three out of four configurations result in negative *ΔQ*_CO_. This indicates that rather than accumulating electrons in CO and thus inducing p-type doping in the sheet, the molecule loses electronic charge, which results in an n-type effect. In contrast, only one of the four values of *ΔQ*_CO_ is negative for the CO@Si-MoTe_2_ system. In this case, the accumulation is comparable to the effect on the pristine sheet. However, the transfer is reduced in comparison.

Interestingly, the distinct differences illustrated between Al and Si may have their origin in the valence configuration of the dopant. The atoms adopt an sp^λ^ hybridization in order to accommodate the trigonal symmetry of X–Mo bonding. Si favors an interaction with the neighboring Mo that has a strong in-plane character (low occupation in p_*z*_ orbitals, high occupation in p_*x*_/p_*y*_, and high *Δh*_X-Te_, values given in Table 1). This suggests a low value of λ and thus hybrids closer in nature to sp^2^. This results in fewer sp-type orbitals needed to be occupied, which in combination with four valence electrons, makes Si less likely to accumulate electrons when interacting with an analyte. This is supported by the values of *ΔQ*_X_^ads^, which show that upon adsorption the dopant loses electronic charge (positive value indicates a hole accumulation) or gains only −0.011 *e* (see Table 3). In comparison, Al adopts hybridization with a somewhat stronger sp^3^ character, which in combination with one fewer electron in Al compared to Si makes the dopant in greater need of electronic accumulation. This is in line with the values of *ΔQ*_X_^ads^ (see Table 3). Regardless of the adsorption configuration of the molecule, Al gains electronic charge, with the value of the transfer up to −0.076 *e*. The large transfer also impacts the Al–Mo bonding. Post-adsorption, the dopant shifts higher relative to Mo suggesting that more electronic charge makes the Al hybrids more sp^3^ in nature.

Contrasting the strong adsorption of CO promoted by Al and Si doping, the P, S, and Cl atoms facilitate a noticeably weaker binding in comparison. Similar to the pristine MoTe_2_, the doped sheets favor a horizontal alignment of the molecule with a limited impact of the C/O orientation relative to the dopant. The strongest interaction is induced by P doping with *E*_ads_ of −234 meV. This is followed by S and Cl, with the values of −156, and −131 meV, respectively. As such, the doping increases the adsorption strength for molecules of CO. However, the effect is relatively limited, as the *E*_ads_ on the pristine sheet reaches values up to −113 meV. This is a result of the structure configuration, where optimization produces comparable geometries regardless of whether the sheet is doped or not (compare Figure 4a with h,j,l, and 4c with i,k,m). The only notable exceptions are the horizontal configurations of CO@P-MoTe_2_, where the C/O–X distances are ∼2.4 Å (horiz-C shown in Figure 4h) compared to 3.66 Å on MoTe_2_ (Figure 4a and b). The reduced separation facilitates an enhanced charge transfer between CO and P-MoTe_2_. The molecule is predicted to accumulate up to −0.128 *e*, and the charge predominantly originates from the depopulation in orbitals of P (compare the values of *ΔQ*_CO_ and *ΔQ*_X_^ads^ given in Table 3). In the case of S and Cl, where the CO–substrate distance is large, the enhanced transfer has not been induced.

**Figure 5:**
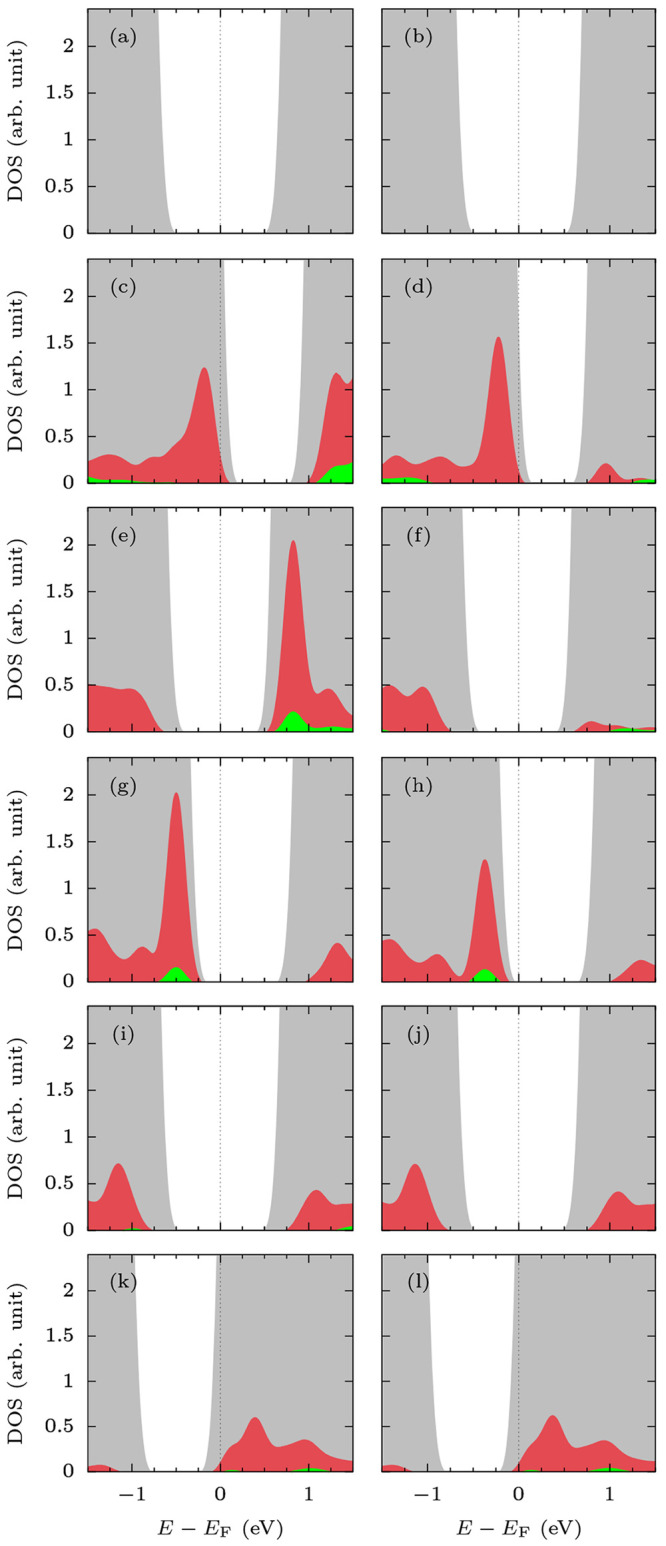
DOS contours of (a,b) MoTe_2_, (c,d) Al-MoTe_2_, (e,f) Si-MoTe_2_, (g,h) P-MoTe_2_, (i,j) S-MoTe_2_, and (k,l) Cl-MoTe_2_. Left-hand-side figures represent preadsorption DOS, while the right-hand-side figures are the DOS post-CO adsorption (the lowest energy configuration). Gray contours represent the total DOS, while red and green the partial DOS of p and s orbitals of X.

Considering the predicted charge transfers, it is important to understand the impact of the molecule–substrate interaction on the electronic properties of the sheets. Table 4 summarizes the values of work function and band gap for CO@MoTe_2_ and CO@X-MoTe_2_, while Figure 5 gives the corresponding DOS. Contours of total DOS of the system and partial DOS of the a and p orbitals of X are shown. Orbital contributions of C and O atoms comprising the CO molecule are not included due to their low density near the Fermi level.

**Table 4:** Work Functions and Band Gaps of Favorable Configurations of CO@MoTe_2_ and CO@X-MoTe_2_

	work function (eV)	band gaps (eV)
MoTe_2_	4.603	1.05
Al-MoTe_2_	4.978	metallic
Si-MoTe_2_	4.614	0.83
P-MoTe_2_	5.042	0.67
S-MoTe_2_	4.712	0.96
Cl-MoTe_2_	4.251	metallic

Figure 5a,b shows the DOS of CO@MoTe_2_ and CO@X-MoTe_2_, respectively. When comparing the contours, it becomes evident that the CO adsorption has virtually no impact on the electronics of the sheet. The adsorption results in only a low hole transfer into MoTe_2_ (see Table 3), which in turn has virtually no impact on its Fermi level, work function, and band gap (see Table 4).

Despite the strong binding of CO, the adsorption on Al-MoTe_2_ gives rise to a relatively low charge transfer (see Table 3), which results in little change in the DOS of the sheet (see Figure 5c and d). The sheet gains electrons in the favorable configuration of CO@Al-MoTe_2_. Hence, the Fermi energy moves up, and the work function drops to 4.978 eV. Adsorption on Si-MoTe_2_ has even less of an effect (see Figure 5e and f). It results in a virtually unchanged work function and band gap (Table 3). Still, the partial DOS of s and p orbitals of Al/Si illustrates a reduced contribution of those orbitals to the unoccupied states near the Fermi level. Those orbitals hybridize with the orbitals of C and move down into the valence band. This represents the filling of the unoccupied sp^3^-type hybrid orbitals via the formation of the fourth Al/Si bond.

In the case of P (Figure 5g,h), S (Figure 5i,j), and Cl doped sheets (Figure 5k,l), the adsorption has a limited impact on the orbitals of the dopants as the interactions with CO are limited to physisorption. The values of charge transfer on S-MoTe_2_ and Cl-MoTe_2_ are low (see Table 3). Both sheets gain holes upon adsorption, which lowers the Fermi energy, and thus increases their work function. However, the changes are small (compare values in Tables 2 and 4). On the other hand, P-MoTe_2_ promotes a significantly enhanced charge transfer of electrons into the molecule (i.e., holes into the sheet; see the values of *ΔQ*_X_^ads^ in Table 3, and the reduced contribution of P orbitals in Figure 5h). The adsorption reduces the band gap to 0.67 eV, notably shifts down the Fermi energy, and increases the work function of the material to 5.032 eV.

### Adsorption of CO_2_ on Pristine and Doped MoTe_2_

In the case of CO_2_ adsorption, this study also employs a number of initial adsorption geometries. Due to the molecule symmetry, three configurations are investigated: (i) CO_2_ placed horizontally with C atom on top of Te/X (horiz-C, Figure S2a, see Supporting Information), (ii) the same but with O atom on top of Te/X (horiz-O, Figure S2b), and (iii) with the molecule placed vertically over Te/X (vert-O, Figure S2c). The analysis follows the same method as outlined for the adsorption of CO, but with the CO_2_ structures replacing those with CO in eqs 2–4.

**Figure 6:**
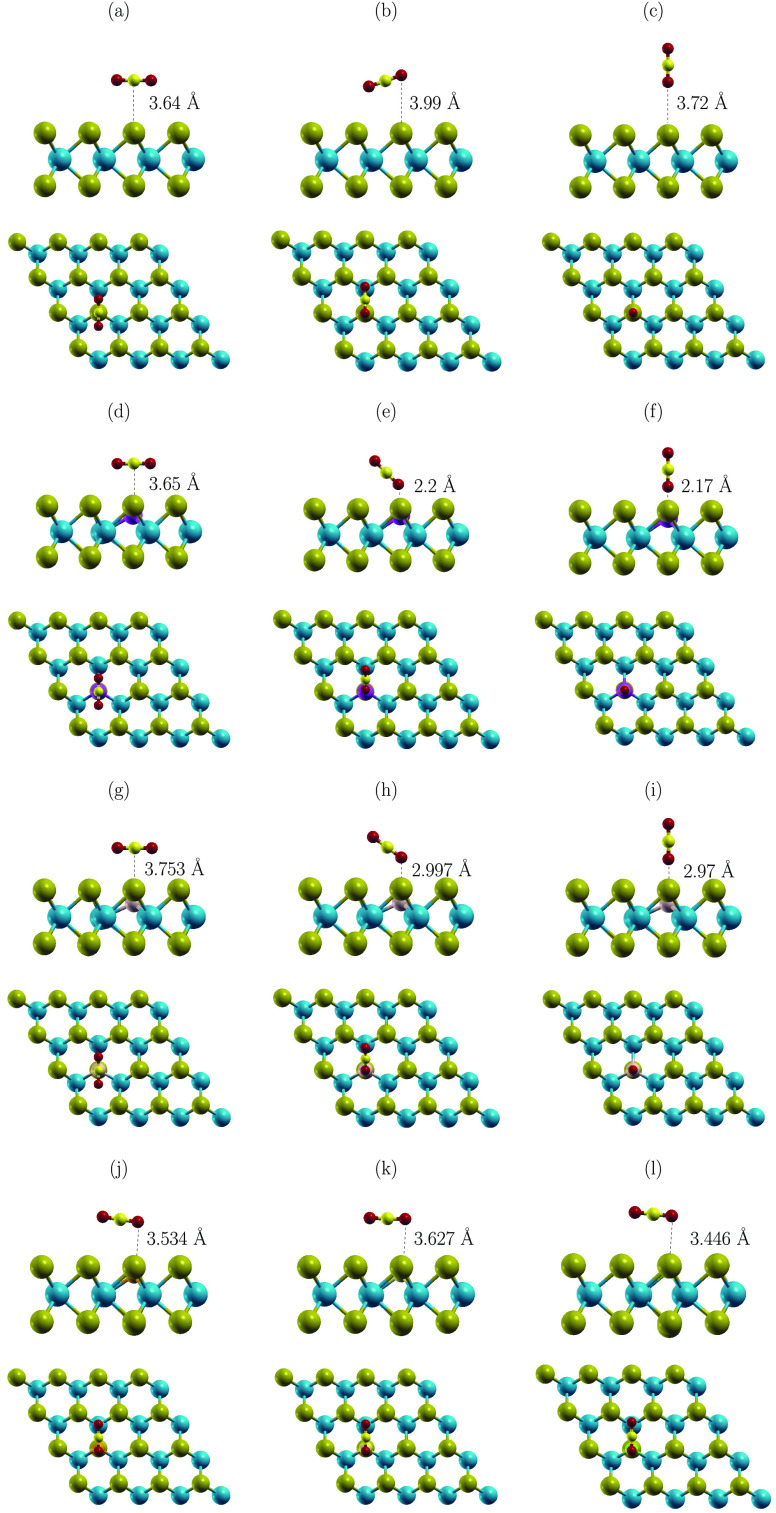
Schematics of optimized structures of CO_2_ on (a) MoTe_2_ (horiz-C), (b) MoTe_2_ (horiz-O), (c) MoTe_2_ (vert-O), (d) Al-MoTe_2_ (horiz-C), (e) Al-MoTe_2_ (horiz-O), (f) Al-MoTe_2_ (vert-O), (g) Si-MoTe_2_ (horiz-C), (h) Si-MoTe_2_ (horiz-O), (i) Si-MoTe_2_ (vert-O), (j) P-MoTe_2_ (horiz-O), (k) S-MoTe_2_ (horiz-O), and (l) Cl-MoTe_2_ (horiz-O).

The adsorption parameters of every investigated structure are summarized in Table 5, while selected configurations are shown in Figure 6. In the case of pristine MoTe_2_, CO_2_ is predicted to have a weak interaction with the substrate. The molecule favors adsorption in horizontal configurations over the vertical with *E*_ads_ of about −150 and −74 meV, respectively. The distances between CO and the neighboring Te are more than 3.5 Å (see Figure 6a–c), which results in a low charge transfer between CO_2_ and the substrate. The molecule accumulates −0.016 and −0.014 *e* when adsorbed horizontally, and only −0.002 *e* in the vertical configuration, which makes the sheet relatively insensitive toward CO_2_. However, the results predict that the interaction strength can be enhanced depending on the dopant.

**Table 5:** Adsorption of CO_2_ on MoTe_2_

	conf	Figure	–*E*_ads_^a^	*Q*_CO2_^b^	*ΔQ*_X_^ads^^c^
MoTe_2_	horiz-C	6a	149	0.016	-
MoTe_2_	horiz-O	6b	146	0.014	-
MoTe_2_	vert-O	6c	74	0.002	-
Al-MoTe_2_	horiz-C	6d	150	0.011	0.034
Al-MoTe_2_	horiz-O	6e	372	–0.111	–0.062
Al-MoTe_2_	vert-O	6f	327	–0.126	–0.059
Si-MoTe_2_	horiz-C	6g	174	0.012	0.021
Si-MoTe_2_	horiz-O	6h	237	–0.005	–0.013
Si-MoTe_2_	vert-O	6i	202	–0.016	–0.021
P-MoTe_2_	horiz-C		213	0.023	0.026
P-MoTe_2_	horiz-O	6j	213	0.011	0.001
P-MoTe_2_	vert-O		110	0.001	–0.032
S-MoTe_2_	horiz-C		214	0.02	0.011
S-MoTe_2_	horiz-O	6k	174	0.012	0.005
S-MoTe_2_	vert-O		99	0.003	–0.017
Cl-MoTe_2_	horiz-C		178	0.016	0.004
Cl-MoTe_2_	horiz-O	6l	159	0.011	–0.001
Cl-MoTe_2_	vert-O		110	0.003	–0.021

a *E*_ads_ is the adsorption energy (meV).

b *ΔQ*_CO_ is the total change in orbital population in CO_2_ (given in *e*).

c *ΔQ*_X_ is the total change in orbital population in X (given in *e*).

In the case of Al-MoTe_2_, the doping promotes the adsorption configurations of CO_2_ with one of the O atoms facing Al. The resulting *E*_ads_ is −372 and −327 meV for horiz-O and vert-O, respectively. Configuration horiz-C gives rise to a weaker interaction (−150 meV), which has a noticeable impact on the relaxed structures. Horiz-C results in the C–Al distance of 3.65 Å (see Figure 6d), while when O faces Al (horiz-O and vert-O); the O–Al separation is only ∼2.2 Å (see Figure 6e and f). This facilitates a more effective charge transfer, where −0.111 (horiz-O), and −0.126 *e* (vert-O) are transferred from the molecule to the substrate, thus n-doping the sheet. The effect is strongly promoted by Al, as approximately half of the transferred charge is accumulated in the dopant (compare the values of *Q*_CO2_ and *ΔQ*_X_^ads^ given in Table 5). In contrast, the doping has virtually no impact on the transfer in the case of horiz-C. Furthermore, the Al doping has very little effect on the adsorption energy and the atomic geometry of the configuration (compare the values in Table 5, and structures given in Figure 6a and d).

Si-MoTe_2_ favors configurations with O facing the dopant. They give rise to adsorption energies of −237 (horiz-O) and −202 meV (vert-O), while the configuration with C facing Si (horiz-C) has *E*_ads_ of −174 meV. As a consequence, the Si doping increases the average strength of CO_2_ adsorption by 81 meV. This is a noticeable enhancement of CO_2_ binding. However, the effect is still less pronounced compared to Al-MoTe_2_ (160 meV). The configurations horiz-O and vert-O result in ∼3 Å separation between O and Si (see Figure 6h and i), which is noticeably shorter than 3.72 and 3.99 Å for the distance O–Te on the pristine sheet (Figure 6b and c), but still significantly longer than ∼2.2 Å predicted between O and Al on Al-MoTe_2_ (Figure 6e and f). Consequently, Si also facilitates n-doping of the sheet. However, the effect is smaller than in the case of Al-MoTe_2_.

The remaining dopants have a limited impact on the adsorption of CO_2_. The relaxed structures are virtually identical to each other. Configurations horiz-C and vert-O are qualitatively similar to those on the pristine sheet. Hence, only horiz-O are shown (Figure 6j–l). The structures favor configurations with C facing the dopant. However, the impact on the adsorption energy is low. On average, the interaction strength is increased by 55 (P), 39 (S), and 26 meV (Cl). Consequently, the dopants have a limited effect on the values of charge transfer.

As a consequence of the low impact of doping on the molecule–substrate interaction, the effects of CO_2_ adsorption on the electronic properties of X-MoTe_2_ are limited (see Figure S3 and Table S1 in Supporting Information). The only notable exception is Al-MoTe_2_. Al doping facilitates a large electron transfer from CO_2_ to the sheet (n-type doping) so that the Fermi level of the sheet moves up and the value of its work function decreases to 4.647 eV.

### Doping-Enhanced Detection and CO/CO_2_ Selectivity

**Figure 7:**
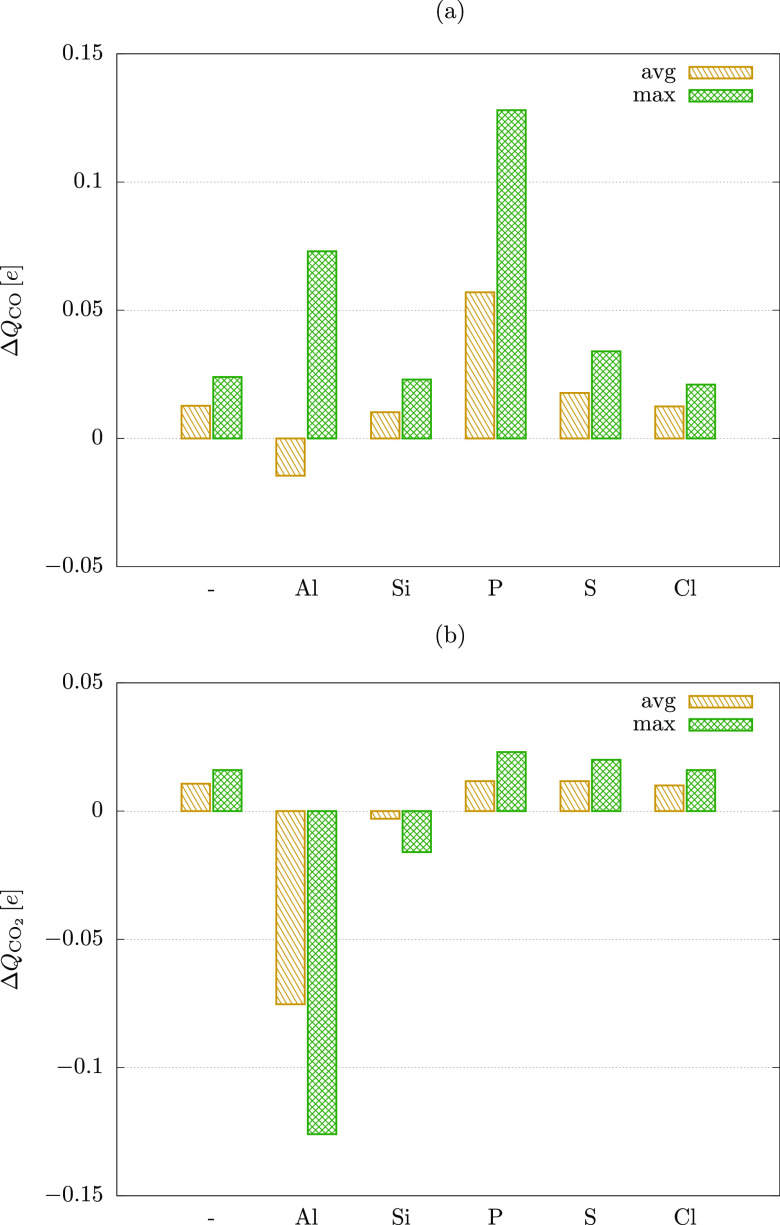
Average and maximum values of charge accumulation in CO (a) and CO_2_ (b) upon adsorption on pristine and doped MoTe_2_.

Considering the low values of adsorption energy, and the dispersive nature of acting forces, the molecule will be able to transition between semistable states even at relatively low temperatures. Hence, the effective charge doping of the substrate, facilitated by the adsorption of the analyte, will result from a spectrum of configurations rather than the accumulated transfers promoted by the lowest-energy geometry. Consequently, Figure 7 shows both average and maximum values of charge transfer *ΔQ*. The former has been calculated as an arithmetic mean of transfers resulting from different configurations. This approach does not account for the geometries with a stronger binding being retained statistically longer, and as they typically result in larger *ΔQ* (see Tables 3 and 5), the true effective transfer should be higher than the average but still lower than the computed maximum. Hence, when analyzed together they should be good indicators of the facilitated effect.

The results given in Figure 7 show that doping can significantly enhance the charge transfer of both molecules. However, the effect is strongly dependent on the dopant and the analyte. In the case of CO (Figure 7a), the adsorption on the pristine sheet results in electron accumulation in the molecule and thus p-doping of MoTe_2_. The average *ΔQ*_CO_ is 0.013 *e*, while the maximum value is 0.024 *e*. Al doping of MoTe_2_ significantly enhances the maximum value of *ΔQ*_CO_ (0.073 *e*), but the average becomes negative (−0.015 *e*). The electron transfer (n-type doping) facilitated by Al is a local effect, and thus it will compete with the intrinsic hole transfer (p-type doping) of CO@MoTe_2_. Hence, this modification of the sheet will likely hamper its sensitivity toward CO, which in combination with the strong binding of the molecule on Al-MoTe_2_ (*E*_ads_ = −957 meV) makes Al not suitable for its detection. All remaining dopants give rise to positive values of *ΔQ*_CO_. However, Si also facilitates strong adsorption of CO (>1 eV), which will greatly impede the recovery of the sensor and thereby hinder its response to changes in CO concentration. Cl doping promotes a weak binding, but the average and maximum values of *ΔQ*_CO_ are only 0.013 and 0.021 *e*, respectively, thus providing no improvement over the pristine sheet.

On the other hand, the interactions with the S doped MoTe_2_ remain weak, while the charge transfer is enhanced. The average and maximum values of *ΔQ*_CO_ are 0.018 and 0.034 *e*, which is a 39% and 43% increase, respectively, over monolayer MoTe_2_. However, S doping has virtually no impact on the type of majority charge carriers in the sheet, while the hole transfer from CO to S-MoTe_2_ facilitates only a minor change in the electrical parameters of the system. In contrast, P-MoTe_2_ retains a relatively weak binding of CO, but the average and maximum transfers are 0.057 and 0.128 *e*, respectively. This represents a substantial (350% and 436%) improvement over MoTe_2_, which in turn has a significant impact on the electrical properties of the sheet. P-MoTe_2_ has been predicted as a p-type semiconductor. Upon adsorption of CO, it is able to accumulate holes more effectively than the pristine MoTe_2_. This notably shifts down the Fermi level, which indicates a large increase in the charge carrier concentration (holes) in the sheet. Consequently, P doping of MoTe_2_ is predicted to be an optimal doping strategy for CO detection.

**Figure 8:**
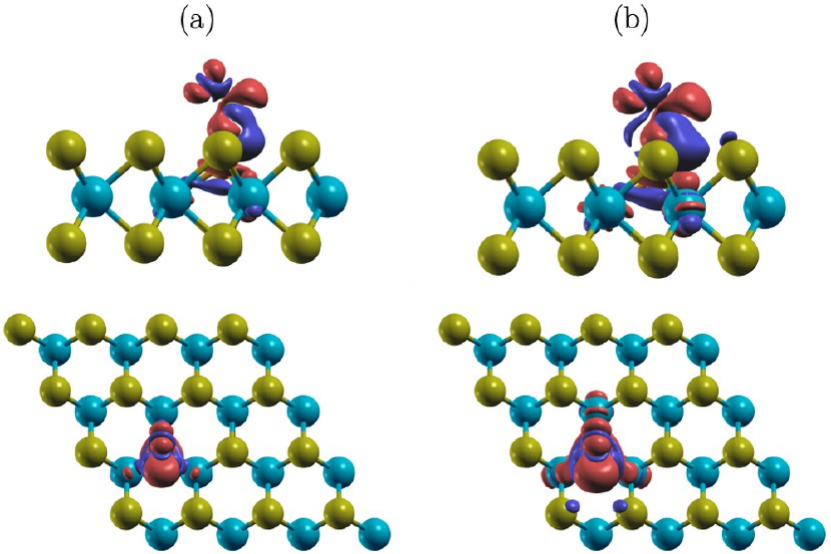
Changes in total electron density after CO adsorbs on P-MoTe_2_. Areas in blue (red) indicate regions of electron depletion (accumulation). The isovalue cutoffs are 0.001 (a) and 0.0005 *e*/au^3^ (b).

The effect predicted for the P-MoTe_2_ is predominantly a product of CO interacting with the phosphorus atom. Figure 8 illustrates the difference in electron density of the favorable configuration of CO@ P-MoTe_2_, given by *δn* = *n*(CO@MoTe_2_) – *n*(CO) – *n*(MoTe_2_), where *n* is the total density of pseudoelectrons. Electron density is seen accumulated on the molecule (color red). The effect is more pronounced for the atom closer to the surface. This results in a noticeable accumulation of charge between C and P, which in part may account for the stronger binding and the large charge transfer. The interaction also results in small electron depletion between C and O (color blue), which is in line with 0.018 Å elongation of the C–O bond upon adsorption facilitated by the electrostatic repulsion resulting from electron accumulation on both atoms. A change in electron density is also present between P and the neighboring Mo, and in some of the Te atoms facing CO, hence making the transfer enhancement a relatively local effect.

P doped MoTe_2_ gives rise to adsorption energies of up to −234 meV compared to −113 meV predicted for the pristine sheet. Hence, in order to ascertain if the stronger interaction will impact the surface diffusion of CO at room temperature, and thus the adsorption site recovery, we have performed a set of three AIMD simulations conducted at 300 K. In each case, the molecule starts adsorbed at the doping site, and all computations produce qualitatively equivalent results. During the AIMD run, CO gradually moves away from its initial adsorption site with ∼9 Å separation after 1.25 ps. This transition occurs predominantly in-plane, as the out-of-plane distance of the molecule increases only slightly in comparison (∼0.5 Å). The simulation predicts that CO can effectively diffuse on the surface of P-MoTe_2_. Hence, P doping should have a limited impact on the adsorption site recovery, and thus should not hamper the response speed of the sensor. Furthermore, as the molecule moves effectively on the surface, the same methods enhancing the recovery rate of MoTe_2_ surfaces should also work for P-MoTe_2_.

**Figure 9:**
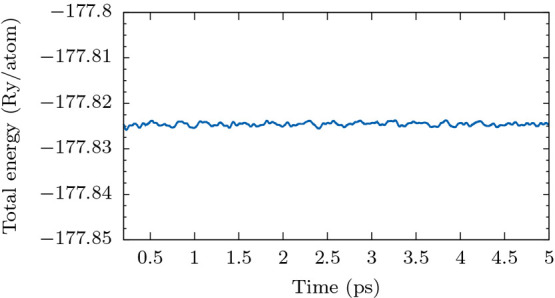
Variation of total energy during molecular dynamics simulation of CO@P-MoTe_2_.

Figure 9 shows the variation of the total energy of CO@P-MoTe_2_ during one of the AMID simulations (the other runs produce qualitatively identical trends). The mean and standard deviation of the total energy were computed to be −177.825 Ry/atom and 3.922 × 10^–4^ Ry/atom, respectively. The drift in the total energy (given by the slope of the linear fit) was found to be 2.091 × 10^–8^ Ry/atom-fs.

Figure 8b shows that doping can significantly impact the charge transfer for CO_2_. However, the effects do not correlate with the changes facilitated by CO (compare the values of *ΔQ* given in Figure 8a and b). The adsorption of CO_2_ on the pristine sheet results in electron accumulation in the molecule, and thus p-doping of MoTe_2_. The average *ΔQ*_CO2_ is 0.011 *e*, while the maximum value is 0.016 *e*. Al doping greatly affects the charge transfer. However, both the average and maximum values of *ΔQ*_CO2_ are predicted to be negative (−0.075 and −0.126 *e*). Thus, the effect does not enhance the intrinsic hole transfer of the pristine sheet but competes with it. The same is also true for Si doping, but the effect is less pronounced, as the average and maximum values of *ΔQ*_CO2_ are −0.003 and −0.016 *e*, respectively. On the other hand, Cl and S doping have virtually no impact on the charge transfer between CO_2_ and the substrate (both have an average *ΔQ*_CO2_ of ∼ – 0.01 *e*). Only P-MoTe_2_ shows some positive effects of doping. The average and maximum values of charge transfer are 0.012 and 0.023 *e*, which is a 9% and 44% improvement, respectively. Hence, P doped sheets should be more sensitive toward CO_2_. However, this has only a low impact on the electronic properties of the sheet when compared to the effects facilitated by CO. Considering that combustion products contain a mixture of CO and CO_2_ (the ratio depends on the oxidation effectiveness), the selective character of the enhanced sensing may prove especially beneficial. The relative response [*ΔQ*_CO_(avg) /*ΔQ*_CO2_(avg)] of P-MoTe_2_ is 5.6 compared to the 1.5 predicted for the pristine sheet. Thus, P doping not only significantly improves the sensing performance of MoTe_2_ toward CO, but also does it with only a small impact on CO_2_ sensitivity, which should allow for more selective detection of CO in CO/CO_2_ mixtures.

This may prove particularly significant for semiconductor-type detectors of CO, i.e., devices where the resistance of the sensing element depends on the concentration of the analyte (e.g., CO). Currently available sensors are made of tin dioxide (SnO_2_), which has to be heated to ∼400 °C for the device to operate. This greatly impacts the power demand of the detector, which (i) reduces their economical viability, (ii) makes them less portable, and (iii) hinders their lifespan. In contrast, it is easier for surface interactions to have a noticeable effect on the carrier concentration of a 2D sheet compared to bulk materials. Thus, a good per-molecule transfer of charge allows 2D sheets to have high sensitivity even at room temperature.^42−45^ Consequently, the low adsorption energy, enhanced charge transfer, and the CO/CO_2_ selectivity of P-MoTe_2_ could make it an effective alternative to SnO_2_.

It is also prudent to note that in some cases, the sensing characteristics of a single TMD sheet can be further enhanced for multilayer systems. It was found that a five-layer structure of MoS_2_ has a several times larger response to NO_2_ than systems with only two layers.^91^ The effect has been correlated with NO_2_ intercalation.^51^ However, for it to be favorable, species have to adsorb strongly and/or require low expansion. This could be particularly beneficial for CO sensing by MoTe_2_ as the sheets promote weaker interlayer interactions compared to MoS_2_. Furthermore, CO is smaller than CO_2_. Hence, its intercalation could (in principle) occur more frequently than CO_2_. However, further studies on the interlayer interactions of doped MoTe_2_ and their intercalation are required to ascertain the potential use of doped multilayer systems.

## Conclusions

The presented work investigates, employing the DFT level of theory, the doping of Al, Si, P, S, and Cl atoms into the Te vacancy of MoTe_2_, and its impact on the sensing characteristics for CO and CO_2_. The computations predict that doping can significantly affect the adsorption energy and the charge transfer of both molecules. However, the effects facilitated by the dopants do not correlate between CO and CO_2_. Considering that the optimal doping strategy for gas detection would require an increased value of the charge transfer near the doping site such that, the effect would enhance the intrinsic transfer of the sheet, but without a significant increase in the adsorption energy, only phosphorus was found to be a viable doping agent for the detection of CO. In the case of P-MoTe_2_, the binding of CO is still relatively weak (AIMD runs show that the molecule can effectively diffuse at 300 K), but the average and maximum transfers are increased significantly by 350% and 436%. Furthermore, the relative (CO/CO_2_) response of P doped MoTe_2_ is 5.6 compared to the 1.5 predicted for the pristine sheet. Thus, the doping should allow for more selective detection of CO in CO/CO_2_ mixtures. Consequently, the low adsorption energy, enhanced charge transfer, and the CO/CO_2_ selectivity of P-MoTe_2_ could make it an effective alternative to currently used sensing materials.

## Supporting Information Available

Initial configurations of CO and CO_2_ before relaxation. Density of state plots of CO_2_@MoTe_2_ and CO_2_@X-MoTe_2_. The values of work function and band gap of CO_2_@MoTe_2_ and CO_2_@X-MoTe_2_MoTe_2_. (PDF)
