# Supplementary material for: Selective Detection of Carbon Monoxide on P-Block Doped Monolayers of MoTe2
Source: ACS Sens. 2022 Jan 19;7(1):272–85. doi: 10.1021/acssensors.1c02246 (PMC8805155; doi:10.1021/acssensors.1c02246)
Supplement: Supplementary file 1 — se1c02246_si_001.pdf [file se1c02246_si_001.pdf]

Selective detection of carbon monoxide on p-block doped monolayers of $MoTe_2$

Maciej J. Szary,* Dominik M. Florjan, and Jakub A. Bąbelek

Institute of Physics, Poznan University of Technology, ul. Piotrowo 3, 61-138 Poznan, Poland

E-mail: maciej.szary@put.poznan.pl

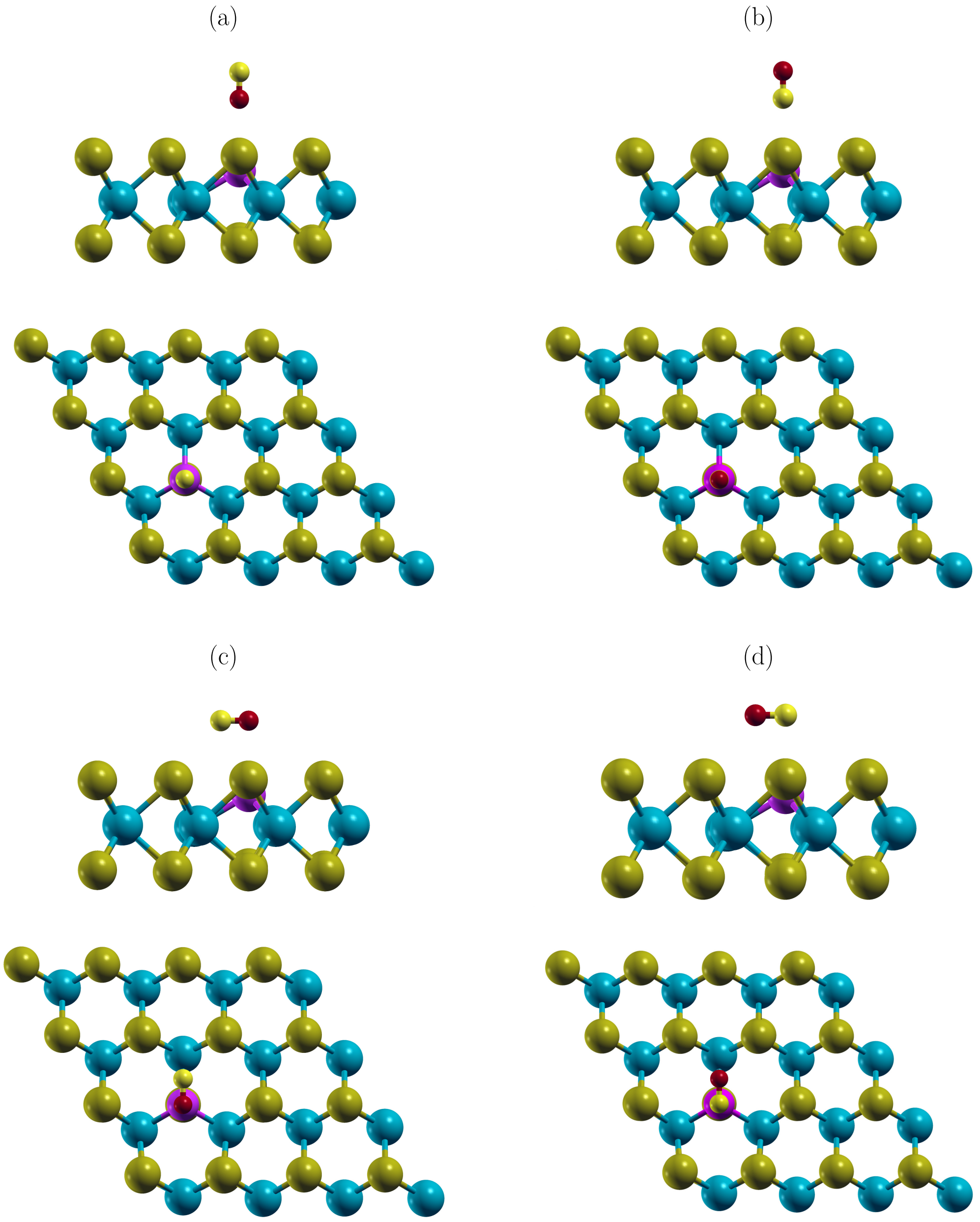

Figure S1: Initial configurations of CO before relaxation: (a) vertical O over the dopant (vert-O), (b) vertical C over the dopant (vert-C), (c) horizontal O over the dopant (horiz-O), and (d) horizontal C over the dopant (horiz-C).

Table S1: Work functions and band gaps of CO_2@$MoTe_2$ and CO@X-$MoTe_2$

	work function (eV)	band gaps (eV)
$MoTe_2$	4.590	1.05
Al-$MoTe_2$	4.647	metallic
Si-$MoTe_2$	4.571	0.83
P-$MoTe_2$	4.884	0.90
S-$MoTe_2$	4.587	0.97
Cl-$MoTe_2$	4.329	metallic

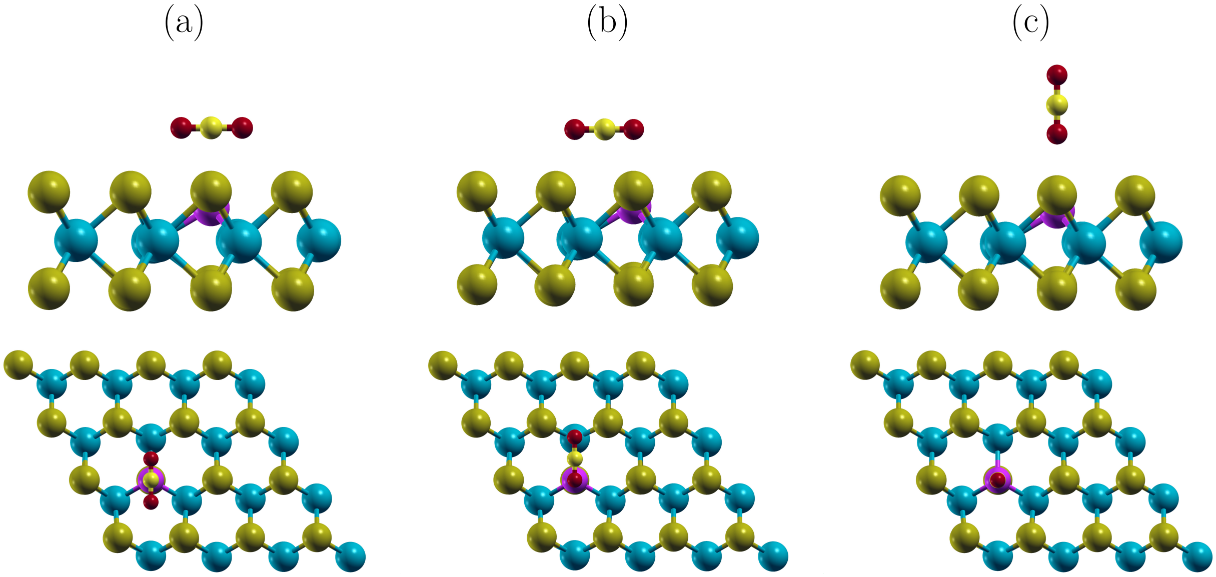

Figure S2: Initial configurations of CO_2 before relaxation: (a) horizontal C on top of X, (b) horizontal O on top of X, and (c) vertical C on top of X.

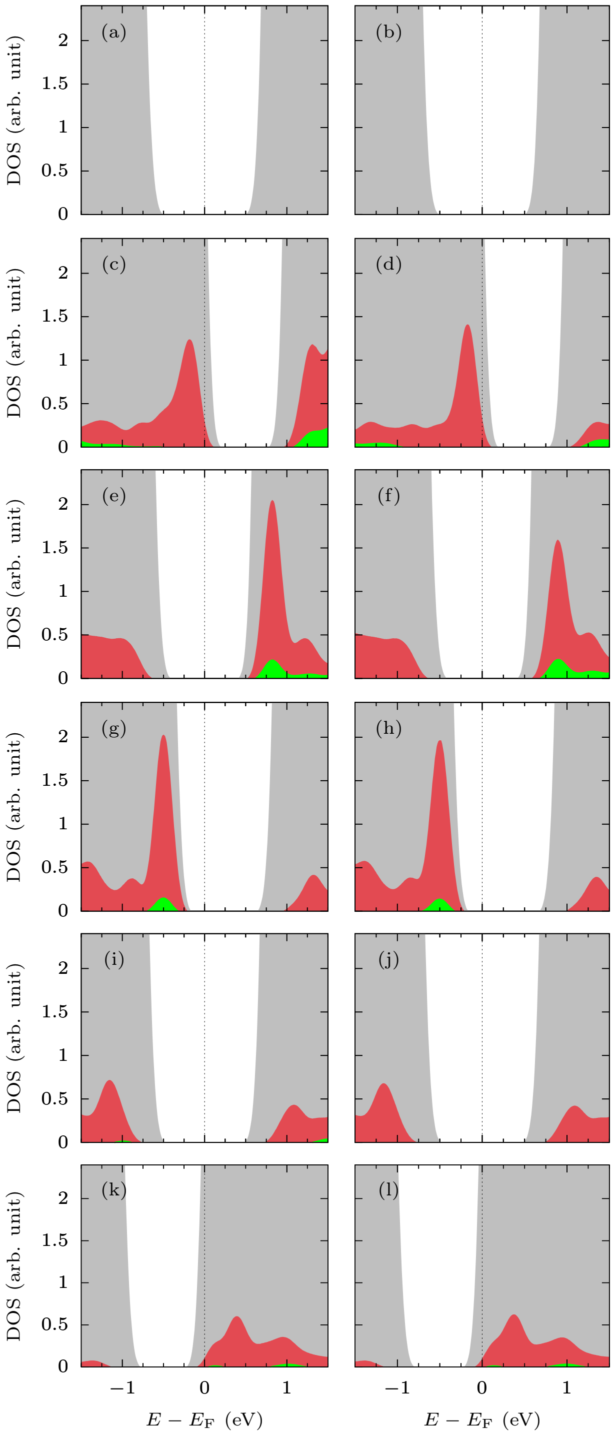

Figure S3: DOS contours of (a,b) $MoTe_2$, (c,d) Al-$MoTe_2$, (e,f) Si-$MoTe_2$, (g,h) P-$MoTe_2$, (i,j) S-$MoTe_2$, and (k,l) Cl-$MoTe_2$. Left hand side figures represent pre-adsorption DOS, while the right hand side figures the DOS post CO adsorption (the lowest energy configuration). Gray contours represent the total DOS, while red and green the partial DOS of p and s orbitals of X.
